# Anastomotic leakage with abscess: Neglected severe complication of bariatric surgery for obesity: A case report

**DOI:** 10.1016/j.ijscr.2019.02.010

**Published:** 2019-02-12

**Authors:** Qing He, Ning-Ning Yang, Yong-Jiu Xie, Yong-Song Guan

**Affiliations:** Department of Oncology, West China Hospital of Sichuan University, Chengdu, Sichuan Province, China

**Keywords:** Bariatric surgery, Complication, Anastomotic leakage, Abscess, Perioperative diet

## Abstract

**Introduction:**

Presently, bariatric surgery is a widespread treatment for obesity and its co-morbidities. Comprehensive evidence from outcomes of bariatric surgery supported that the surgery is safe and effective. However, we should be aware of the multiple risks of bariatric surgery, especially the severe infection at the operation site.

**Case presentation:**

A 30-year-old woman was hospitalized for anastomotic leakage with abscess after laparoscopic sleeve gastrectomy. After 53 days of percutaneous drainage, absolute diet fasting, parenteral nutrition and culture based antibiotics etc., she was discharged for recovery.

**Discussion:**

Theoretically, malabsorption and early diet will increase the risk of anastomotic leakage, but this problem is seldom to be mentioned in related articles. Moreover, it seems to be common that people received bariatric surgery had a relatively short post-operative stay.

**Conclusion:**

Essential nutritional support, longer post-operative hospital stay and strict follow-up are necessary to minimize this complication.

## Highlights

• Nowadays, postoperative stay is too short for bariatric operation.
• Nowadays, postoperative nutritional support is insufficient.
• Post-operative fever is the sign of poor healing or anastomotic leakage.

## 1 Introduction

Bariatric surgery is currently thought to be the most effective therapeutic option for obese patients. However, the method carries substantial risks, including postoperative malabsorption, hormonal disturbance, infection in surgical site, etc. There is no consensus on perioperative diet and parenteral nutrition that should be crucial for the wound healing process which is often incomplete at the time of discharge. Theoretically, malabsorption and early diet will increase the risk of anastomotic leakage, but this problem is seldom to be mentioned in related articles. Herein, we report a case of anastomotic leakage with abscess after laparoscopic sleeve gastrectomy for morbid obesity and discuss the possible reasons to call attention to the risk of anastomotic leakage after bariatric surgeries.

The work has been reported in line with the SCARE criteria and the related guidelines have been cited in the references [1].

## 2 Case presentation

**Fig. 1:**
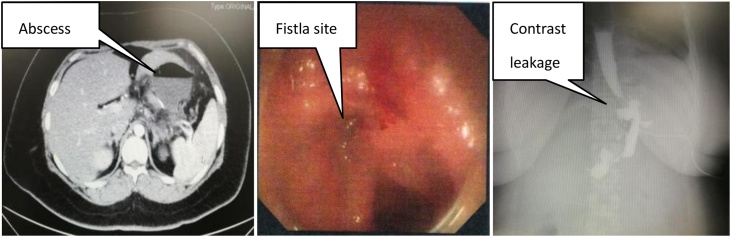
The CT, gastroscopy and esophagography revealed that the presence of anastomotic leakage and abscess beside the stomach.

A 30-year-old woman presented for bariatric surgery. Her body mass index(BMI) was 41.7. She was discharged only 3 days after the laparoscopic sleeve gastrectomy. For the abrupt loss of appetite, the patient only took pure warm water from the next day of the surgery for a week, then, clear liquid diet for another week till she was hospitalized again because of fever (around 38°C), low degree dull abdominal pain and vomiting on the 13th post-operative day. Routine blood test suggested an infection profile. The abdominal computed tomography (CT), esophagography and gastroscopy revealed the presence of abscess and related anastomotic leakage (Fig. 1).

## 3 Management

Absolute diet fasting and parenteral nutrition were prescribed. Then, percutaneous drainage of abscess was performed and isthmus group streptococcus was detected in the culture test of pus. Subsequently, an antibiotic was prescribed.

After 53-day drainage, CT and following esophagography confirmed that the abscess was disappeared and no signs of leakage existed. As everything went well, the patient was discharged 5 days later. 5 months following, no signs of recurrence were found.

## 4 Discussion

Generally, surgical site infection is correlated to obese status, nutritional condition, diabetes, smoking history and pre-operation inpatient time. Due to the bactericidal effect of gastric acid, gastric abscess is rare to see after gastric surgeries. Because of the high mortality rate of gastric abscess (37%–100%), the gastric resection will only be considered on the condition of no alleviation of fever and it is very dangerous to treat the patients with anastomotic leakage by medical treatment alone [2]. Fortunately, the outcome of our patient was favorable under percutaneous drainage and antibiotics etc. Former reports showed that the pathogens usually come from the oral microflora, which was confirmed in our case [3].

Laparoscopic operation is a newly-developed minimally-invasive operation. Compared to traditional surgery, laparoscopic operation is superior in minor wounds and much shorter inpatient days. Some studies suggested that sleeve gastrectomy might be the safest method compared with gastric bypass and gastric banding surgery [4]. However, it seems that the stapling device used in the bariatric surgery is not as reliable as the surgeons think. The distance between two stapler teeth may be different from each other for various reasons and the area where the longer tooth distance exists needs a longer time to heal and has higher risk of leakage. So, more strict check after device sewing and manual additional sewing in the weak area may be of help to decrease the risk of leakage after surgeries. To minor side effects, bariatric artery embolization (bariatric embolization of arteries for the treatment of obesity) was used on obesity patients and similar effect was achieved more safely. However, compared to the surgery, embolization does not decrease the volume of stomach and the stomach may regain its blood supply sooner or later depends on the embolization agents. So, further researches are needed to evaluate whether the embolization is as effective as the surgery [5].

We should be aware of the multiple risks of bariatric surgery, especially the severe infection and anastomotic leakage at the operation site. Too short post-operative hospital stay plus early diet should be extremely dangerous for these patients. However, it seems to be common that people received bariatric surgery had a relatively short post-operative stay. For example, a investigation held in UK found that the average post-operative stay of people received bariatric surgery was 2.7 days, and only 3% of the patients remained in hospital more than 5 days [6].

When and to what extent, the postoperative patients take their diet is another question. Until now, there is no consensus on how to deal with this condition. Obviously, sufficient nutrition support will help the stomach to heal while too early diet is not; however, the patient described in our case seemed to take her diet (pure warm water) too early (from the next day of the surgery) while sufficient nutrition support was neglected (no parenteral nutrition was prescribed during the whole initial hospital stay). Longer post-operative stay, later diet and essential parenteral nutrition should be more suitable for these patients.

## 5 Conclusion

Overall, laparoscopic bariatric operation is safe and effective. However, the surgeon, the internist, and the patient must be aware of the multiple risks of this kind of surgery, especially the severe infection and anastomotic leakage at the operation site. Essential nutritional support and strict follow-up are crucial to minimize these complications. Moreover, we should be aware that the post-operative fever, no matter to what degree, may be the sign of anastomotic leakage or poor healing of the stomach.

## Conflicts of interest

No conflicts of interest.

## Sources of funding

We had no sources of funding for this case report.

## Ethical approval

This case is exempt from ethnical approval.

## Consent

We have obtained written and signed consent to publish a case report from the patient.

## Author contribution

Qing He, Ning-Ning Yang and Yong-Jiu Xie drafted the manuscript, Yong-Song Guan revised it critically.

## Registration of research studies

This case is a retrospective study.

## Guarantor

Qing He.

## Provenance and peer review

Not commissioned, externally peer-reviewed.
